# The Impact of Environmental Pollution and Economic Growth on Public Health: Evidence From China

**DOI:** 10.3389/fpubh.2022.861157

**Published:** 2022-03-28

**Authors:** Xiaochun Zhao, Mei Jiang, Wei Zhang

**Affiliations:** ^1^School of Management, Anhui University, Hefei, China; ^2^School of Public Administration, Sichuan University, Chengdu, China

**Keywords:** environmental pollution, economic growth, public health, panel data, China

## Abstract

A comprehensive understanding of the impact of economic growth and environmental pollution on public health is crucial to the sustainable development of public health. In this paper, an individual fixed effect model is used to analyze the impact of environmental pollution and economic growth on public health, based on the panel data of 30 provinces in China from 2007 to 2018. The research finds that: First, the health status of China's four regions is not only affected by economic growth and environmental pollution, but also affected by the per capita disposable income and urbanization rate. Second, there is a long-term balanced relationship between China's economic growth, environmental pollution and public health. Third, environmental pollution harms children's health and significantly increases the perinatal mortality, while economic growth helps to reduce the perinatal mortality. Fourth, environmental pollution plays a regulatory role between economic growth and public health. Fifth, there are significant regional differences in the impact of environmental pollution and economic growth on public health. Among them, the degree of harm caused by sulfur dioxide emissions on mortality in northeastern China is significantly higher than that of the eastern China, eastern China is higher than that of the western China, and western China is higher than that of the central China. Finally, in order to reduce the adverse consequences of environmental pollution on public health in the process of economic development, this study puts forward relevant policy suggestions.

## Introduction

In recent years, China's economy has undergone earth-shaking changes and people's living standards have also been significantly improved. From 2007 to 2015, China's gross domestic product (GDP) increased by 2.6 times. At the same time, the total amount of industrial waste gas emission also increased by 1.8 times. It can be seen that with the rapid development of economy, environmental problems are becoming more and more prominent (1). Environmental pollution can cause great harm to people's health and bring huge social losses (2, 3). According to the statistics from the World Health Organization (WHO), air pollution can produce a large amount of toxic gases, which impair children's development ability and lead to chronic diseases such as respiratory tract infections. Among them, 93% of children under the age of 15 suffer invisible harms. Therefore, accurate grasp of relationship between environmental pollution, economic development and public health has important reference value to improve people's health. As early as the 1970s, foreign scholars analyzed the impact of environmental pollution on public health based on Grossman's health production function (4). It has been recognized that there is a stable and balanced relationship between environmental pollution and public health, and environmental pollution has had a huge negative effect on public health and increased the burden of medical expenditures (5). At present, China is the largest energy consumer, and the healthcare needs caused by environmental pollution are increasing day by day (6). To cope with this severe challenge, the Chinese government has complied the Outline of the Healthy China 2030 Plan, emphasizing that we should focus on improving people's health so as to meet people's growing health needs and improve health equity. However, the basis for this goal lies in the comprehensive understanding of health influencing factors. It is necessary to further clarify the impact of regional economic development and environmental pollution on public health in China, which is of great significance to promote the development of regional public health. Therefore, this paper takes China as an example, establishes a regression model based on the Grossman health production function, and conducts an empirical analysis on the relationship between China's economic development, environmental pollution and public health, as well as the differences in health levels in various regions, so as to put forward relevant policy suggestions and provide intellectual support for the steady development of public health.

The next arrangement of this paper is as follows: the second part sorts out the relevant research results of environmental pollution, economic development and public health, and proposes the research innovation points of this paper; the third part mainly includes research methods and data sources; the fourth part is the research results that show the main findings of this research, and the fifth part concludes this research, puts forward corresponding policy recommendations, and summarizes the shortcomings of this research.

## Literature Review

At present, the research on the correlation among environmental pollution, economic growth and public health has received extensive attention from academic circles. The existing literature focuses on the relationship between environmental pollution and economic growth, economic growth and public health, environmental pollution and public health. In terms of the relationship between environmental pollution and economic growth, some scholars made quantitative analysis through methods of data envelopment analysis (DEA), Granger causality test analysis and Environmental Kuznets Curve (EKC) hypothesis from the perspective of green economic development. For example, Zhang et al. used the super efficiency general direction distance function model to measure the efficiency of resource-saving cities based on the panel data of 197 cities in China from 2011 to 2015, and found that the rapid development of urban economy often comes at the expense of environmental pollution, thus putting forward relevant suggestions for the sustainable development of green economy (7). Jiang et al. analyzed the causal relationship between economic policy uncertainty and environmental pollution in the United States, and found that there is a significant causality between them (8). Based on 284 prefecture-level panel data, Chang et al. studied the EKC hypothesis using the spatial dynamic panel data model and proposed that the relationship between environmental pollution and economic growth in China conforms to an inverted U-shaped curve, and features robustness (9). In terms of the economic growth and public health, a two-way relationship is found after combing the literature. On the one hand, economic development has an impact on public health. On the other hand, public health has an impact on economic development. Guhn et al. analyzed the relationship between economic level and children's health status by using the Canadian database on health care and economic development, and found that there is a significant positive correlation between the two, so that poverty is considered as one of the main reason cause public health problems (10). Wang et al. conducted an empirical analysis of 31 provinces in China by established an individual fixed effect model, and found that the target setting of economic growth and public health quality took on a *U*-shaped trend (11). Long et al. analyzed the relationship between environmental pollution and health level by establishing a health damage model and found that the lower the health level, the greater the economic loss (12). From the perspective of environmental pollution and public health, academia has conducted a lot of research on the impact of environmental pollution on public health and achieved certain results. After reviewing the literature, it is found that scholars' research generally starts from two aspects: analytical method and econometric model. First, for analytical method, scholars generally adopted dose-response relationship and exposure response principle. Klepac et al. analyzed the consequences of pregnancy through air pollution indicators and found that exposure to ambient air pollutants throughout pregnancy is positively correlated with preterm birth (13), and environmental pollution has adverse effects on pregnant women; Manisalidis et al. analyzed the exposure to pharmaceuticals method, found that the elderly, children and asthma patients are more susceptible to the effects of high concentrations of environmental pollution, and there are short-term and long-term effects, thus increasing the mortality rate (14). Second, for econometric model, scholars generally conduct research on the basis of the Grossman health production function. Hao et al. used the data of Chinese provinces from 1998 to 2015 to analyze the relationship between environmental pollution and medical expenditure by using Gaussian Mixture Model (GMM), and found that the higher the degree of environmental pollution, the higher the frequency of residents seeking medical treatment, and the higher the health expenditure (15). It can be seen that there is a negative correlation between environmental pollution and public health level. Qu et al. used the Stochastic Impacts by Regression on Population, Affluence, and Technology model (STIRPAT) to show that haze pollution relates to the decrease of public health level, while the per capita disposable income and medical service level relate to the increase of public health level and thereby help reduce mortality, so the importance of environmental governance is emphasized and relevant suggestions are put forward (16).

Overall, the existing literature systematically discusses the relationship between environmental pollution, economic growth and public health, but there is still room for improvement. First, the direct impact of economic growth on environmental pollution can indirectly compromise public health, so it is necessary to analyze the impact of economic growth and environmental pollution on public health. Second, public health levels in different regions vary a lot due to the different levels of regional economic development. Most of the existing studies focus more on a certain region, and less on the overall picture of China and its sub-regions. Therefore, this paper uses the panel data of 30 provinces in China from 2007 to 2018, establishes an individual fixed effect model based on the Grossman health production function, and divides China into four regions, namely the eastern region, the northeastern region, the central region and the western region. Research on the relationship between environmental pollution, economic growth and public health in various regions aims to provide policy recommendations for health equity, as well as experience and reference for public health in other developing countries.

## Research Methods and Data Sources

### Panel Data Model

#### Unit Root Test

In the process of panel data regression analysis, in order to ensure the stability of the research data, it is necessary to perform a unit root test on the panel data, so as to avoid false regression in the data (17, 18). If it passes the test, it means that the data is stationary and there is no unit root; if it does not pass the test, the data needs to be differentiated to eliminate the unit root and change to a stationary sequence. Therefore, the following auto regressive model is established:


(1) $$\begin{array}{l} {\text{y}}_{\text{it}}=x_{\text{it}}\alpha_{i}+\rho_{i}{\text{y}}_{\text{it}-1}+\varepsilon_{\text{it}},i=1,2,...,N,t=1,2,...,T_{i} \end{array}$$

Where *x*_*it*_ represents the exogenous variable vector in the model, ε_*it*_ represents the independent and identically distributed random error term, and ρ_*i*_ is the auto regressive coefficient. If|ρ_*i*_*|* = 1, it means that there is a unit root, that is, the model is not stationary, if |ρ_*i*_*|* < 1, it means that the model does not have a unit root, that is, the model is stable, *N* represents the number of cross-sectional data, and *T*_*i*_ represents the t-th period of the i-th cross-sectional data.

Panel data unit root test can be divided into long panel test and short panel test according to the total number of sections and periods. Long panel unit root test includes Levin-Lin-Chu Test (LLC Test), Im-Pesaran-Shin Test (IPS Test), Breitung Test and Augmented Dickey-Fuller Fisher Test (ADF Fisher Test);short panel unit root test includes Harris-Tzavalis Test (HT Test). This paper mainly adopts the HT test applicable to short panels.

#### Co-integration Test

The panel data co-integration test is based on the same-order single integration in order to test whether there is a long-term stable balanced relationship between variables. Common co-integration tests are divided into two categories: Engle and Granger's two-step (EG two-step method) panel data co-integration test and Johansen's co*-*integration test. This paper mainly uses the Kao co-integration test and the Pedroni co-integration test of the EG two-step method.

#### Model Estimation

The estimation process of the panel data model can be divided into two steps: first, determine the model form. Panel data model can be divided into constant coefficient model, variable intercept model and variable coefficient model according to whether there is individual influence and structural changes; second, describe the model impact. That is, making a decision between random effect model and individual fixed effect model. The basic models of panel data are as follows:


(2) $$\begin{array}{l} \text{Variable coefficient model : }{\text{y}}_{\text{it}}=\alpha_{\text{it}}+{\text{X}}_{\text{it}}\beta_{\text{it}}+{\text{u}}_{\text{it}} \end{array}$$

(3) $$\begin{array}{l} \text{Variable intercept model : }{\text{y}}_{\text{it}}=\alpha_{\text{it}}+{\text{X}}_{\text{it}}\beta +{\text{u}}_{\text{it}}+\text{m} \end{array}$$

(4) $$\begin{array}{l} \text{Constant coefficient model : }{\text{y}}_{\text{it}}=\alpha +{\text{X}}_{\text{it}}\beta +{\text{u}}_{\text{it}} \end{array}$$

There are two main assumptions:


(5) $$\begin{array}{l} H_{1}\beta_{1}=\beta_{2}=\ldots =\beta_{N}\\ H_{2}\alpha_{1}=\alpha_{2}=\ldots =\alpha_{N},\beta_{1}=\beta_{2}=\ldots =\beta_{N} \end{array}$$

Through the Fisher-test (F-test), if the alternative hypothesis *H*_2_ is accepted, it conforms to the constant coefficient model, select Equation (4), and the test ends. If the alternative hypothesis *H*_2_ is rejected, the original *H*_1_ shall be tested. If *H*_1_ is accepted, it conforms to the variable intercept model and select Equation (3). Otherwise, if *H*_1_ is rejected, it conforms to the variable coefficient model and select Equation (2). After determining the model form, the Hausman test can be used to judge whether to build a solid effect model or a random effect model.

#### Model Construction

In order to study the relationship between economic development, environmental pollution and public health, this paper is based on the Grossman health production function, and draws on the research results of Lu et al. (19) to add economic factors and pollution factors into the model. The regression model is as follows:


(6) $$\begin{array}{l} PM_{it}=\alpha_{1}InSO_{2it}+\alpha_{2}InPGDP_{it}+\alpha_{3}NHP_{it}+\alpha_{4}PMHS_{it}\\ \;+\alpha_{5}InPDI_{it}+\alpha_{6}UR_{it}+\alpha_{7}PAR_{it}+C_{it}+\varepsilon_{it} \end{array}$$

The explanatory variable is the level of public health. According to WHO's measure of health, this paper selects perinatal mortality (*PM*) as an indicator of public health.

The core explanatory variables are economic development and environmental pollution. The level of economic development is measured by per capita gross domestic product (*PGDP*) (20). Compared with other environmental pollution, air pollution is more harmful to human body (21, 22), so this paper selects sulfur dioxide emission (*SO*_2_) as the environmental pollution indicator.

The control variables are medical service conditions, residents' living standards and demographic characteristics. The indicators of medical service conditions include the number of health personnel (*NHP*) and the price of medical and health services (*PMHS*). The indicators of residents' living standards include per capita disposable income (*PDI*) and urbanization rate (*UR*) (23). The population aging rate (*PAR*) was used as the index of population characteristics.

### Data Sources

This paper uses the national data from 2007 to 2018 as the research sample for empirical analysis. Relevant data are compiled from China Statistical Yearbook (2008–2019), China Health Statistical Yearbook (2008–2019), and China Environmental Statistical Yearbook (2008–2019). In order to eliminate the heteroscedasticity of data, the logarithm of SO_2_, per capita GDP and per capita disposable income is taken.

## Analysis Results

### Descriptive Analysis

The economic development status of each province in China is different, and the degree of environmental pollution is also significantly different (24), so the health level of each province is also different. In order to better understand the public health level of various regions in China, This paper divided 30 provinces into eastern region, northeast region, central region and western region according to its geographical location (see Table 1).

**Table 1:** Descriptive statistical analysis results.

**Variable**	**Eastern China**				**Northeastern China**			
	**Mean**	**Std. dev**.	**Min**	**Max**	**Mean**	**Std. dev**.	**Min**	**Max**
*PM*	0.0051	0.002	0.002	0.009	0.0079	0.002	0.005	0.012
*SO_2_*	543,330.7	513,319.4	2,672	1,827,397	541,210	331,123	89,586	1,234,000
ln*(SO_2_)*	12.482	1.500	7.891	14.418	13.006	0.665	11.403	14.026
*PGDP*	64,620.07	29,021.72	14,476.69	140,761.30	41,491.24	13,407.51	18,475.42	65,424.51
ln*(PGDP)*	10.967	0.487	9.580	11.855	10.576	0.354	9.824	11.089
*NHP*	6.457	2.417	2.750	15.460	5.513	0.668	4.160	7.000
*PMHS*	1.0268	0.025	0.984	1.154	1.037	0.031	1.006	1.111
*PDI*	31,021.58	12,306.25	10,996.87	68,033.60	28,995.51	47,306.23	10,245.28	301,717.90
ln*(PDI)*	10.266	0.396	9.305	11.128	9.977	0.573	9.235	12.617
*UR*	0.662	0.141	0.403	0.896	0.588	0.049	0.532	0.681
*PAR*	0.099	0.024	0.016	0.152	0.105	0.018	0.077	0.150
**Variable**	**Central China**				**Western China**			
	**Mean**	**Std. dev**.	**Min**	**Max**	**Mean**	**Std. dev**.	**Min**	**Max**
*PM*	0.0056	0.002	0.0024	0.011	0.0083	0.004	0.003	0.019
*SO_2_*	697,823.7	393,378	120,808	1,564,000	597,474.8	374,222.9	46,471	1,456,000
ln*(SO_2_)*	13.276	0.642	11.702	14.263	13.043	0.801	10.747	14.191
*PGDP*	32,791.88	12,346.78	12,036.86	66,531.27	32,830.43	15,473.61	7,286.842	71,936.91
ln*(PGDP)*	10.321	0.409	9.396	11.105	10.281	0.506	8.894	11.184
*NHP*	4.637	1.159	2.620	6.900	5.044	1.439	2.140	8.500
*PMHS*	1.0271	0.020	1.001	1.106	1.028	0.022	0.988	1.125
*PDI*	21,762.18	7,759.428	1,656.70	39,385.80	21,271.18	7,212.269	10,012.34	38,304.70
ln*(PDI)*	9.907	0.458	7.413	10.581	9.904	0.357	9.212	10.553
*UR*	0.483	0.061	0.343	0.603	0.466	0.082	0.282	0.655
*PAR*	0.098	0.014	0.073	0.132	0.137	0.443	0.055	5.152

As mentioned above, in order to eliminate the impact of data heteroscedasticity, this paper takes the logarithm of SO_2_, per capita GDP, and per capita disposable income to conduct descriptive analysis of each indicator. From the perspective of public health, the average value of perinatal mortality in the eastern region is the lowest, while the average value of perinatal mortality in the western region is the highest, which are 0.0051 and 0.0083, respectively. At the same time, the minimum value of mortality is 0.002 and the maximum value is 0.019, indicating that there are significant differences in the health levels among provinces in China. In terms of environmental pollution, the average value of SO_2_ in the eastern region is the lowest, while that in the central region is the highest, which are 12.482 and 13.276, respectively. From the perspective of economic growth, the average value of per capita GDP in the western region is the lowest, which is 10.281, while the average value in the eastern region is the highest, which is 10.967.For other control variables, the number of health workers, medical prices, per capita disposable income and urbanization rate in the eastern region are better than those in other regions. The region with the lowest average proportion of people over 65 is the central region, while The mean values of the control variables in the western region are all lower. To sum up, the central region has the highest sulfur dioxide emission, but the mortality is not the highest. It can be seen that the health status of various regions in China is also affected by other factors in the process of environmental pollution.

### Panel Data Stability Test

Since the units of each variable are different, in order to narrow the dimensional gap between the data and facilitate subsequent regression calculations, it is necessary to standardize all the variables after taking the logarithm of some data. At the same time, in the process of panel regression, in order to avoid unstable variables and false regression, it is essential to test the unit root of the data to ensure the effectiveness of the data (25). Since this paper uses the data of 30 provinces in China from 2007 to 2018, which is a short panel, and the number of cross-sectional data is larger than the number of time series, so the HT test for short panel data is used for unit root test (see Table 2). The original sequence has unit roots and is a non-stationary sequence. Due to the volatility of the data, the difference method is used to eliminate the unit root. As can be seen from Table 2, each data passes the HT test under a confidence level lower than 1%, rejecting the original hypothesis, that is, the data after the first-order difference is stable and there is no unit root. However, all variables are stable only after the first-order difference, that is, the first-order single integration accumulates force, it still needs to be co-integrated test whether there is a long-term stable equilibrium relationship between the observed variables.

**Table 2:** Unit root test.

**Variable**	**HT TEST**			
	**Statistic**	**H_**0**_**	***P*-value**	**Outcome**
Δz*PM*	−0.1043	Panels contain unit roots	0.0000^***^	Smooth
Δzln*(SO_2_)*	0.0094	Panels contain unit roots	0.0000^***^	Smooth
Δzln*(PGDP)*	0.3925	Panels contain unit roots	0.0000^***^	Smooth
Δz*NHP*	−0.6910	Panels contain unit roots	0.0000^***^	Smooth
Δz*PMHS*	−0.3638	Panels contain unit roots	0.0000^***^	Smooth
Δzln*(PDI)*	−0.4800	Panels contain unit roots	0.0000^***^	Smooth
Δz*UR*	−0.1425	Panels contain unit roots	0.0000^***^	Smooth
Δz*PAR*	−1.2241	Panels contain unit roots	0.0000^***^	Smooth

z represents for standardization. *p < 0.1, **p < 0.05,

*** *p < 0.01*.

### Panel Data Co-integration Test

Co-integration test methods include EG two-step method and Johansen co-integration test method. In this paper, the Pedroni test suitable for heterogenous test and Kao test for same root test in EG two-step method are mainly used. The null hypothesis is that there is no co-integration test. As can be seen from Table 3 that the national panel data rejects the null hypothesis under the Kao test and the Pedroni test, that is, through the co-integration test, each explanatory variable and the explained variable have a long-term stable equilibrium relationship, the data volatility is small, and the pseudo regression phenomenon is avoided. Therefore, the next research can be carried out.

**Table 3:** Co-integration test.

**Test**	**Statistical indicators**	**Statistics**	***P*-value**
Pedroni	Modified Phillips-Perron t	9.6325	0.0000^***^
	Phillips-Perron t	−10.5118	0.0000^***^
	Augmented Dickey-Fuller t	−9.6681	0.0000^***^
Kao	Modified Dickey-Fuller t	−2.9579	0.0015^***^
	Dickey-Fuller t	−6.2983	0.0000^***^
	Augmented Dickey-Fuller t	−2.2853	0.0111^**^
	Unadjusted modified Dickey	−4.5845	0.0000^***^
	Unadjusted Dickey-Fuller t	−6.9964	0.0000^***^

*p < 0.1,

** *p < 0.05*,

*** *p < 0.01*.

### Model Setting Test

There are generally three types of model estimation methods for panel data, including mixed regression models, fixed-effects models and random-effects models (26). As for which model is suitable for the data of sulfur dioxide, per capita GDP and two core explanatory variables, three steps are needed to test. First, the F-test is conducted. The original hypothesis is the selected mixed regression model, and the alternative hypothesis is the selected fixed effect model. From the test results, the f statistical values of the three models are 73.02, 80.09, and 83.79, respectively, and the null hypothesis is rejected at the significance level of 1%.So, the fixed effect model is selected. Second, through Lagrange Multiplier Test (LM Test), it can be seen from Table 4 that the three models reject the null hypothesis at the significance level of 1%, that is, they refuse to establish a mixed regression model and choose a random effect model. Third, Hausman test is performed to determine whether to choose a random effect model or a fixed effect model. The null hypothesis of the Hausman test is to choose the random effect model, and the alternative hypothesis is to choose the individual fixed effect model. According to the test results, both the sulfur dioxide model and the total model reject the original hypothesis at the significance level of 1%, The model of per capita GDP rejects the original hypothesis at the 5% significance level, that is, the individual fixed effect model should be selected for the following empirical analysis.

**Table 4:** Model setting test.

**Test method**		**H_**0**_**	**Statistical indicators**	**Statistics**	***P*-value**	**Outcome**
*SO_2_* data model	F Test	Choose mixed regression	F(29, 324)	73.02	0.0000	Reject mixed regression, choose fixed effect model
	LM Test	Choose mixed regression	chibar2(01)	1,073.03	0.0000	Reject mixed regression model and choose random effect model
	Hausman Test	Choose random regression	chi2(7)	36.90	0.0001	Reject random effect model, choose fixed effect model
*PGDP* date model	F Test	Choose mixed regression	F(29, 324)	80.09	0.0000	Reject mixed regression, choose fixed effect model
	LM Test	Choose mixed regression	chibar2(01)	1,201.35	0.0000	Reject mixed regression model and choose random effect model
	Hausman Test	Choose random regression	chi2(7)	18.39	0.0103	Reject random effect model, choose fixed effect model
Total data model	F Test	Choose mixed regression	F(29, 323)	83.79	0.0000	Reject mixed regression, choose fixed effect model
	LM Test	Choose mixed regression	chibar2(01)	1,209.08	0.0000	Reject mixed regression model and choose random effect model
	Hausman Test	Choose random regression	chi2(7)	23.11	0.0032	Reject random effect model, choose fixed effect model

### Panel Data Regression Results

According to Equation (6), an individual fixed effect model is constructed for regression analysis to obtain the results (see Table 5).

**Table 5:** Results of national empirical analysis (2007–2018).

**Variable**	**(1)**	**(2)**	**(3)**	**(4)**	**(5)**	**(6)**
	**z** ***PM***	**z** ***PM***	**z** ***PM***	**z** ***PM***	**z** ***PM***	**z** ***PM***
zln*(SO_2_)*	1.532^***^−12.94	0.196^*^ −1.94			0.465^***^−4.53	0.456^***^ −4.56
zln*(PGDP)*			−0.755^***^ (−33.13)	−0.427^***^ (−5.97)	−0.869^***^ (−25.96)	−0.546^***^ (−7.36)
z*NHP*		0.012 −0.21		−0.027 (−0.50)		−0.031 (−0.59)
z*PMHS*		0.005 −0.27		−0.001 (−0.04)		−0.002 (−0.15)
zln*(PDI)*		−0.189^***^ (−5.79)		−0.090^**^ (−2.59)		−0.076^**^ (−2.25)
z*UR*		−1.194^***^ (−8.99)		−0.447^***^ (−2.73)		−0.456^***^ (−2.87)
z*PAR*		0.001 −0.07		0.001 −0.04		−0.002 (−0.11)
_cons	−0.000 (−0.00)	0.000 (0.00)	−0.000 (−0.00)	−0.000 (−0.00)	−0.000 (−0.00)	−0.000 (−0.00)
*N*	360	360	360	360	360	360
*R* ^2^	0.277	0.74	0.748	0.763	0.762	0.776
*F*-statistic	167.423^***^	175.707^***^	1,097.788^***^	198.036^***^	591.708^***^	183.082^***^

*t-statistics in parentheses*.

* *p < 0.1*,

** *p < 0.05*,

*** *p < 0.01*.

From Table 5, the regression results of national panel data show that the F statistics of column (2), column (4) and column (6) pass the test at the significance level of 1%, and *R*^2^ are relatively high, which are 0.740, 0.763, and 0.776, respectively, indicating that the fitting effect of the three columns is very good. On the whole, Comparing the results of column 2, column 4 and column 6, it can be found that the fit of column 6 is better. Column (1) also shows that there is a positive correlation between SO_2_ and perinatal mortality. Sulfur dioxide emission has passed the significance level test of 1%, and the regression coefficient is 1.532, which means that for every 1% increase in SO_2_, the perinatal mortality will increased by 1.532%, that is, environmental pollution has a negative impact on the public health level of residents. Comparing column (1) and column (2), the impact of SO_2_ on perinatal mortality has decreased from 1.532 to 0.196% after the introduction of other control variables, and passed the significance level test of 10%, proving that other control variables help to reduce the impact of environmental pollution on public health. From the column (3), the per capita GDP passed the test at the 1% significance level with a coefficient of −0.755, which indicates that the growth of per capita GDP has a promoting effect on perinatal mortality. For every 1% growth of per capita GDP, the perinatal mortality will decrease by 0.755%, that is, there is a significant negative correlation between economic growth and health status. Column (3) obtains column (4) after introducing other control variables, and its coefficient increases from −0.755 to −0.427, which demonstrates that other control variables are not conducive to the impact of economic growth on residents' public health level. According to the results of column (6), the two core explanatory variables passed the test at the significance level of 1%, with coefficients of 0.456 and −0.546, respectively. Among the control variables, per capita disposable income passed the significance level test of 5%, with a coefficient of −0.076, declaring that per capita disposable income has a positive effect on health status. The urbanization rate is also significantly negatively correlated with the perinatal mortality, that is, it has passed the significance level of 1%, with a coefficient of −0.456, which is better than the impact of per capita disposable income on the perinatal mortality. The main reason is that the urbanization rate has increased, the education level of residents has improved, and their attitudes have changed accordingly, which is conducive to their investment and attention to health (27). Other control variables, namely health personnel, medical price and aging rate, have no significant impact on perinatal mortality. The reason may be that with the improvement of socio-economic level (15), the impact of individual demand on health level has increased compared with social supply (28). Comparing column (2) with column (6), by introducing the variable of per capita GDP, the sulfur dioxide emission coefficient increases from 0.196 to 0.456, which illustrates that under the condition of economic growth, environmental pollution is increasing and the health level is decreasing.

### Heterogeneity Analysis

Due to the health levels of the 30 provinces in China are different. In order to understand the status of each region more clearly, this paper divides the 30 provinces in China into four regions, establishes a solid effect model for heterogeneity analysis, and tests the results. The results are shown in Table 6.

**Table 6:** Heterogeneity analysis results.

**Variable**	**Eastern China**	**Northeastern China**	**Central China**	**Western China**
	**zPM**	**zPM**	**zPM**	**zPM**
zln*(SO2)*	1.011^***^−6.21	1.536^***^ −6.29	0.560^*^−1.96	0.745^***^ −4.6
zln*(PGDP)*	−0.594^***^ (−3.56)	−0.462^***^ (−4.13)	−0.615^***^ (−3.78)	−0.717^***^ (−4.18)
z*NHP*	−0.036 (−0.99)	−0.663^**^ (−2.53)	0.532^***^−2.75	0.31 −1.64
z*PMHS*	0.013 −0.81	−0.016 (−0.61)	0.016 −0.52	0.004 −0.12
zln*(PDI)*	0.038 −0.34	0.028 −0.8	−0.018 (−0.48)	−1.141^***^ (−6.37)
z*UR*	−0.821^***^ (−5.85)	−1.156^***^ (−4.14)	−0.892^**^ (−2.48)	1.083^*^ −1.7
z*PAR*	0.957^***^−3.6	0.936 −1.12	−0.524 (−0.38)	−0.006 (−0.33)
_cons	0.486^***^−5.01	0.089 −0.6	−0.887^***^ (−8.83)	0.891^***^ −2.93
*N*	120	36	72	132
*R* ^2^	0.855	0.947	0.856	0.848
*F-*statistic	102.375^***^	90.985^***^	62.226^***^	107.195^***^

*t-statistics in parentheses*.

* *p < 0.1*,

** *p < 0.05*,

*** *p < 0.01*.

Comparing the regression results of the four regions, the health status of the four regions is significantly affected by SO_2_ and per capita GDP. From the SO_2_ emission indicators, the coefficients of each region are 1.011, 1.536, 0.560, and 0.745, respectively. It is observed that the environmental pollution in Northeast China has the greatest impact on the health level, that is, for every 1% increase in sulfur dioxide, the perinatal mortality will increase by 1.536%. From the variable of per capita GDP, all regions passed the test at the significance level of 1%. Among them, the health status of the western region is more likely to be affected by economic growth. The main reason is that the economic development level of the western region is low, and people tend to consume expenditure and ignore service expenditure (29), Therefore, economic growth is conducive to enhancing the service expenditure and health level in the western region.

In terms of regions, the urbanization level and the aging rate in the eastern region have pasted the test under the significance level of 1%, with coefficients of −0.821 and 0.957, respectively, showing that the urbanization rate and mortality in the eastern region are negatively correlated, while the aging rate and mortality are positively correlated. This is primarily due to the rapid economic development and high urbanization rate in the eastern region (30), it is conducive to increasing residents' investment in public health (31), while the eastern region has a high aging rate and greater demand for health. Every 1% increase in the number of health workers in Northeast China will reduce the mortality by 0.663%, and pass the significance test of 5%. The basic reason is that sulfur dioxide emission has great impact on health status (32, 33). Therefore, increasing the number of health workers can promote the health level. The coefficient of health population in the central region is 0.532, which is significantly positively correlated with mortality. This is contrary to the hypothesis. The main reason is that the population density in the central region is low, and the per capita health population may be greater than that in other regions (34), while the low level of economic development promotes the high mortality. The western region is greatly affected by people's disposable income, and the higher the per capita disposable income, the lower the mortality. This is because the western region is restricted by the level of economic development and the residents have a lower tendency to spend money on medical services (35). To sum up, the health level of various regions in China is affected by different factors, and there are significant regional differences. Therefore, it is indispensable to develop public health services according to local conditions.

## Conclusion and Discussion

Based on the panel data of 30 provinces in China from 2007 to 2018, this paper uses the individual fixed effect model to conduct regression analysis and heterogeneity analysis, and to explore the impact of regional economic growth and environmental pollution on public health. From the study, we can find that: First, the health status of various regions in China is significantly affected by other factors in addition to the variables of environmental pollution and economic growth. For example, per capita disposable income and urbanization rate are negatively correlated with mortality, per capita disposable income is significant at 5% level, and urbanization rate is significant at 10% level. Second, there is a long-term and stable equilibrium relationship between China's economic growth, environmental pollution and public health. Third, SO_2_ and perinatal mortality are significantly positively correlated, and have passed the significance test of 1%. For every 1% increase in sulfur dioxide emissions, the mortality increase by 0.456%. The per capita GDP has a positive effect on the reduction of perinatal mortality. If the per capita GDP increases by 1%, the perinatal mortality will decrease by 0.546%.Besides, the health level is also significantly affected by two control variables, namely per capita disposable income and urbanization rate. Fourth, environmental pollution affects the impact of economic growth on public health. As can be seen from Table 5, after introducing the variable of sulfur dioxide emission, the coefficient of total per capita domestic production changes from −0.427 to −0.546. Fifth, there are significant regional differences in the health level of the four regions and they are affected by different factors. In Northeast China, environmental pollution has the greatest impact on public health, while in Western China, the economic development level has the greatest impact on public health. Based on the above conclusions, in order to better promote the level of public health, the following policy suggestions are put forward:

First, the government should focus not only on economic growth, but also on environmental pollution and public health (36). Above all, the government should shift the mode of economic development, develop an environment-friendly society, and promote sustainable economic development. Moreover, the government should increase capital investment to promote the optimization and upgrading of industrial structure and increase support for new energy industry, so as to reduce the impact on the environment in the process of economic development. Eventually, the government should increase health care expenditure and further promote the infrastructure of medical service. Second, enterprises should clarify their responsibility of environmental protection and accelerate the application of energy-saving technologies (37). While enterprises have contributed to the economic growth, due to the external effects in the development of market economy, they have caused significant harm to the environment at the same time. Therefore, it is necessary to clear corporate social responsibility (38). Third, the four regions should adopt policies according to local conditions to improve the level of public health. The eastern region should pay Attention to the negative impact of aging population on public health, the northeast and central regions should focus on the construction of medical infrastructure and increase the number of health personnel, and the western region should accelerate economic development to promote the level of public health.

The empirical research conducted in this study helps to promote China's public health level, but this study can be further improved from the following aspects: Firstly, this paper adopts panel regression method to analyze the impact of economic development and environmental pollution on public health, other econometric methods can be used to study the relationship between regional economic development, environmental pollution and public health (39, 40). Secondly, there are many environmental pollution variables affecting public health. This paper only considers the variable of air pollution. Therefore, considering the impact of other factors on publihc health is a research direction in the future.

## Data Availability Statement

The raw data supporting the conclusions of this article will be made available by the authors, without undue reservation.

## Author Contributions

XZ and MJ: Methodology. XZ: writing—original draft preparation. WZ: writing—review and editing. All authors have read and agreed to the published version of the manuscript.

## Conflict of Interest

The authors declare that the research was conducted in the absence of any commercial or financial relationships that could be construed as a potential conflict of interest.

## Publisher's Note

All claims expressed in this article are solely those of the authors and do not necessarily represent those of their affiliated organizations, or those of the publisher, the editors and the reviewers. Any product that may be evaluated in this article, or claim that may be made by its manufacturer, is not guaranteed or endorsed by the publisher.
